# The regulatory role and mechanism of exosomes in hepatic fibrosis

**DOI:** 10.3389/fphar.2023.1284742

**Published:** 2023-12-01

**Authors:** Youli Yao, Da Chen, Zengchang Yue

**Affiliations:** ^1^ College of Electronics and Information Engineering, Shandong University of Science and Technology, Qingdao, China; ^2^ Department of Neurology, Mindong Hospital Affiliated to Fujian Medical University, Ningde, China

**Keywords:** exosomes, hepatic fibrosis, signaling pathways, hepatic stellate cells, therapy targets

## Abstract

Globally, the prevalence and fatality rates of liver disorders are on the rise. Among chronic liver conditions, hepatic fibrosis stands out as a central pathological process. Despite this, approved treatments for hepatic fibrosis are currently lacking. Exosomes, small extracellular vesicles secreted by various cell types, play a significant role in intercellular communication and have emerged as essential mediators in liver fibrosis. In this regard, this review compiles the mechanisms through which exosomes regulate hepatic fibrosis, encompassing diverse targets and signaling pathways. Furthermore, it delves into the regulatory impact of exosomes modulated by natural plant-derived, endogenous, and synthetic compounds as potential therapeutic strategies for addressing hepatic fibrosis.

## 1 Introduction

Hepatic fibrosis, marked by the excessive buildup of extracellular matrix proteins, signifies a progressive liver ailment capable of culminating in cirrhosis and liver failure (Gines et al., 2021). Hence, prioritizing the treatment of hepatic fibrosis before its progression to cirrhosis or liver failure becomes critical.

The liver is an important energy metabolism organ that participates in processes such as glucose metabolism and lipid metabolism (Rui, 2014). Hepatic fibrosis is often accompanied by metabolic disorders. Exosomes can serve as messengers, transmitting information including metabolic regulation, such as miRNA, thereby affecting the regulation of energy balance in the liver (Kim and Hartig, 2022). In recent years, the role of exosomes, small extracellular vesicles, in various physiological and pathological processes has gained significant attention. Exosomes are secreted by various cell types and serve as potent mediators of intercellular communication (Isaac et al., 2021). These nanosized vesicles are rich in proteins, lipids, and nucleic acids, allowing them to transfer biological information between cells (Gurunathan et al., 2021). Exosomes have been implicated in the pathogenesis of diverse ailments, including cardiovascular disorders, metabolic conditions, and immune-related maladies. An expanding body of research is dedicated to exploring the potential of exosomes for both diagnosing and managing a range of medical conditions (Kalluri and LeBleu, 2020; Gurunathan et al., 2021).

**FIGURE 1:**
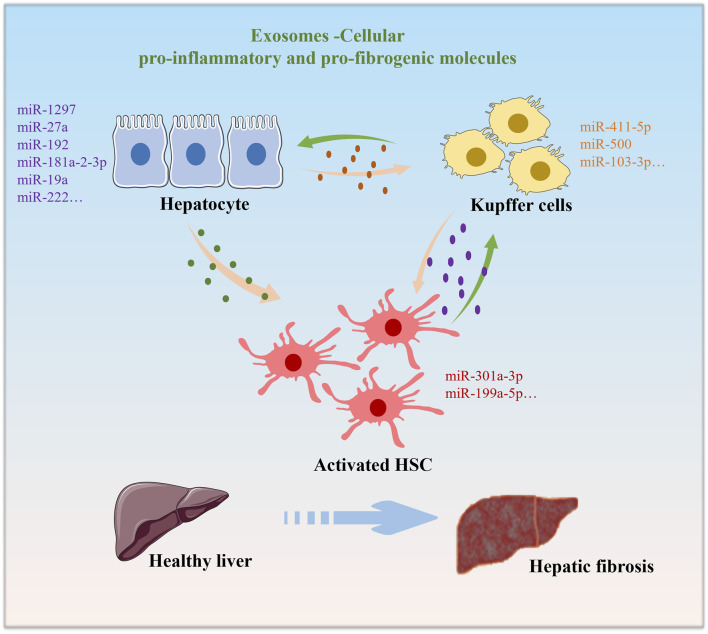
Under normal physiological conditions, Exosomes are cross-communication strategy between various cell types. Exosomes derived from hepatocytes, macrophages, or HSCs upon external stimuli exposure alter the functions of each other, leading to the progression of hepatic fibrosis.

Some studies have shown that extracellular vesicles released by liver cells may carry information about lipid metabolism. This can affect the storage and decomposition of lipids, thereby affecting energy balance. Extracellular vesicles can participate in cell regeneration and repair processes in the liver (Jiao et al., 2021). This is crucial for restoring liver function and maintaining energy balance, especially when liver damage occurs. The release of extracellular vesicles may play a role in the development of chronic liver disease (Shi et al., 2021). They can transmit inflammatory signals and affect the metabolic state of the liver (Shi et al., 2021). This further highlights the relationship between extracellular vesicles and energy balance in the liver. Various cell types, including hepatocytes, hepatic stellate cells (HSCs), and immune cells, contribute exosomes that exert influence over energy metabolism during hepatic fibrosis (Figure 1). Abnormal energy metabolism is one of the pathological factors leading to hepatic fibrosis (Karthikeyan et al., 2016). The activation of HSCs is a central event in the occurrence and development of hepatic fibrosis, which requires energy (Aydin and Akcali, 2018). For the energy metabolism of HSCs in l hepatic fibrosis, many targets and signaling pathways are involved in the activation process of extracellular vesicles and HSCs. The key pathways or targets of phosphatidylinositol 3-kinase (PI3K)/AKT/and AMP-activated protein kinase (AMPK) serve as energy state sensors and participate in the regulation of liver fibrosis energy metabolism by extracellular vesicles.

In summary, extracellular vesicles play various roles in the liver, including intercellular communication, metabolic regulation, repair, and disease progression. These roles are closely related to the energy balance of the liver, as the liver is one of the main energy metabolism organs. Therefore, studying the function and role of extracellular vesicles in the liver helps to better understand the energy balance of the liver and the mechanisms of related metabolic diseases, and also provides important insights for potential treatment strategies.

The precise mechanisms by which exosomes modulate energy metabolism in hepatic fibrosis are still under investigation. Within this review, we consolidate the significance of exosomes in the progression of hepatic fibrosis within this context. Moreover, we discuss the modulatory effect of exosomes by natural plant-derived, endogenous and synthetic compounds for the treatment of hepatic fibrosis.

## 2 Crucial targets and signaling cascades of exosomes implicated in hepatic fibrosis

### 2.1 PI3K/AKT

The PI3K pathway is a critical signaling cascade involved in cell growth, survival, and metabolism. Exosomes, as mediators of intercellular communication, can transfer bioactive molecules, including proteins, microRNAs, and lipids, that may activate PI3K in recipient cells. In the lipotoxic hepatocyte, exosomal miR-1297 can promote the activation of HSC and accelerate the progression of hepatic fibrosis via activation of the PI3K/AKT signaling pathway (Luo et al., 2021a). The liver sinusoidal endothelial cells secrete exosomal SphK1, which promote AKT activation and promote HSCs migration. These exosomes contain various molecules related to fibrosis, such as growth factors, extracellular matrix proteins, etc. In addition, molecules in exosomes may also affect the survival and function of liver cells by activating the PI3K/AKT pathway. These molecules can be transmitted between various cells in the liver through exosomes, thereby affecting the process of liver fibrosis (Sung et al., 2018). Hepatocytes and liver sinusoidal endothelial cells interact with HSCs through exosomes, in turn regulating hepatic fibrosis (Sung et al., 2018). Therefore, exosomes interact with the PI3K/AKT pathway affect the progression of hepatic fibrosis.

### 2.2 PINK1/parkin

Mitochondria are essential for energy production and play a central role in regulating cellular metabolism. When cells experience stress, such as oxidative stress, damage to mitochondria can occur. Exosomes may participate in cellular stress responses, and there is some evidence that they can transport mitochondrial components or signals related to mitochondrial health or damage and influence mitochondrial function (Konaka et al., 2023; Kong et al., 2023; Li et al., 2023; Picca et al., 2023). Mitophagy refers to the selective clearance of damaged mitochondria by cells through the mechanism of autophagy to protect cells. Mitophagy failure is reported to occur in steatotic or fibrotic livers (Kim et al., 2015; Meira Martins et al., 2015). The phosphatase and tensin homolog-induced kinase 1 (PINK1) is the central overseer of the mitophagy pathway (Kang et al., 2016). PINK1, the key target of exosomal miR-27a, primarily mediates mitophagy. Exosomal miR-27a was released from lipotoxic hepatocytes and inhibit mitophagy and promote hepatic fibrosis by negatively regulating PINK1 expression (Luo et al., 2021b). Specifically, through an *in vitro* primary cell culture experiment that mimicked proximal communication between hepatocytes and HSCs, exosomal miR-27a were demonstrated to be preferentially and rapidly taken up by activated HSCs.

### 2.3 AMPK/ULK1

AMP-activated protein kinase, often referred to as AMPK, is an enzyme that plays a crucial role in cellular energy homeostasis and metabolism. AMPK signaling system is an energy sensor that regulates the metabolism of organisms, cells, and lipids (Garcia and Shaw, 2017; Yang et al., 2020). This energy sensor, AMPK, upholds cellular metabolism to ensure energy equilibrium (Gu et al., 2020). Through activating autophagy via the AMPK pathway, human umbilical cord mesenchymal stem cells-derived exosomes (hucMSC-EXs) ameliorated hepatic glucose and lipid metabolism, augmenting AMPK/ULK1-mediated autophagy (He et al., 2020; Song et al., 2023). Mesenchymal stem cell-derived exosomes (MSC-Exs) have been considered a novel therapeutic strategy for hepatic fibrosis (Du et al., 2021; Kim et al., 2021). Exosomes from various sources of MSCs typically exhibit similar functionalities (Wei et al., 2023). HucMSC-Ex has been reported to involve in cell survival, immune conditioning, and damage repair (Wei et al., 2023). Other studies have shown that hucMSC-Exs is a promising therapy for IL-6-induced acute hepatic injury (Shao et al., 2020). Additionally, treatment with hucMSC-EXs prompted the activation of the AMPK-mediated PPARα/CPT-1A and the SREBP-1C/FASn signaling pathways (Yang et al., 2023).

## 3 Modulation of exosome for the treatment of hepatic fibrosis

Manipulating intercellular exosome communication holds the potential to disrupt hepatic fibrosis through the modulation of diverse targets and signaling cascades. This section compiles recent research focusing on compounds that exhibit the ability to regulate exosome activity, offering potential avenues for the therapeutic management of hepatic fibrosis and presenting novel concepts for both scientific investigation and clinical exploration.

### 3.1 Exosome modulation by natural plant-derived compounds

This section underscored the modulation of exosome by natural plant-derived compounds for hepatic fibrosis therapy. Phillygenin is a bioactive compound that derives its name from its discovery in the plant Forsythia suspensa, an herb commonly used in traditional Chinese medicine. Conditioned media and exosomes from Phillygenin-treated macrophages were shown to have inhibitory effects on the expression of various factors associated with HSC activation, such as MMP2, TIMP1, TGF-β, α-SMA, COL1, and NF-κB (Ma et al., 2023). The study found that Phillygenin may reduce HSC activation by inhibiting macrophage-derived exosomal miR-125b-5p, which is known to target Stard13. This results in the restoration of Stard13 expression in HSCs, further contributing to the inhibition of HSC activation (Ma et al., 2023). In summary, the research suggests that Phillygenin has the potential to inhibit hepatic fibrosis by modulating macrophage polarization from pro-inflammatory M1 to anti-inflammatory M2 phenotype and by reducing HSC activation (Ma et al., 2023). These effects are mediated through the inhibition of specific signaling pathways and the regulation of miRNA expression in macrophage-derived exosomes (Ma et al., 2023). Derived from Curcuma Longa, curcumin is a potent ingredient known for its robust anti-inflammatory, antioxidant, and antitumorigenic properties (Tawfeek and Kasem, 2023). Curcumin preconditioned mesenchymal stem cells derived exosomes transplantation regulated the genes responsible for fibrogenesis of the liver (Tawfeek and Kasem, 2023). Astaxanthin, a xanthophyll carotenoid with antioxidant properties, which inhibit the activation of HSCs by modulate the exosomal miR-382-5p (Bae et al., 2022). Thymoquinone (TQ), the main active constituent of Nigella sativa seeds, has been found that TQ inhibit the activation of HSCs by modulating the expression of exosomal miR-30a (Geng et al., 2021). Daucosterol is a phytosterol glycoside widely present in Salvia miltiorrhiza Bunge and Rehmannia glutinosa (Gaertn) (Deng et al., 2023). It is reported that Daucosterol can inhibit the migration of hepatocellular carcinoma cells and improve the hepatic fibrosis induced by carbon tetrachloride in mice (Osman et al., 2016; Zeng et al., 2017). Nevertheless, the establishment of data supporting daucosterol’s regulatory mechanisms in liver protection is pending. In a current study, the authors investigated the potential therapeutic effects of daucosterol in the context of liver failure. The study involved a mouse model of hepatic failure and the use of exosomes derived from umbilical cord mesenchymal stem cells (UCMSCs) as a potential treatment. Treatment with exosomes alone or in combination with daucosterol reduced the liver index and lowered levels of liver enzymes, as well as pro-inflammatory factors. Exosome treatment alone or in combination with daucosterol suppressed the mRNA expression levels of IL-6 and reduced STAT3 protein expression in the liver. This research highlights a potential approach for addressing liver failure by combining the hepatoprotective properties of daucosterol with the regenerative potential of UCMSC-derived exosomes. Daucosterol combined with exosomes isolated from primary mouse umbilical cord mesenchymal stem cells can improve hepatic damage induced by lipopolysaccharide/D-galactosamine through regulating the IL-6/STAT3 signaling pathway (Deng et al., 2023). While the study demonstrates the beneficial effects of daucosterol and UCMSC-derived exosomes in ameliorating liver damage and inflammation in a mouse model, it does not delve deeply into the underlying molecular mechanisms that govern these effects. Additional research is needed to uncover the precise mechanisms through which daucosterol exerts its hepatoprotective properties. Astragalus total saponins (AST) and glycyrrhetinic acid (GA) are the main components of Astragalus and licorice, respectively (Deng et al., 2022). Zhou et al. found that AST combined with GA can significantly inhibit the activation of HSCs by affecting the exosomes of macrophages, thereby inhibiting hepatic fibrosis (Deng et al., 2022). In this study, exosomes derived from normal macrophages were isolated. The results showed that co-culturing LPS-induced macrophage exosomes with JS1 cells notably increased the expression of Collagen-1 (Col-1) and Alpha smooth muscle actin (α-SMA) in JS1 cells. However, pretreatment with AST combined with GA showed a significant reversal effect. Further analysis indicated that the levels of phosphorylated (p)-Smad2 and p-Smad3 in JS1 cells significantly increased after the macrophages were induced with LPS. In contrast, pretreatment with AST + GA significantly decreased the levels of p-Smad2 and p-Smad3. This research explores a new mechanism for the anti-hepatic fibrosis effects of the traditional Chinese medicine components found in the Huangqi Decoction, focusing on the role of exosomes. Salidroside, a bioactive compound derived from various plant sources such as Rhodiola rosea. Salidroside‘s biological functions has been widely studied. It is well known that it has features like anti-fibrotic, anti-inflammatory, and antioxidant properties. Research indicated that salidroside can hinder hepatic fibrosis through reducing the exosomal SphK1-induced activation and migration of HSCs (Ye et al., 2021). These compounds derived from plants exhibit the ability to regulate the biogenesis, content selection, and release of extracellular vesicles, which has an impact on regulating liver fibrosis related diseases.

### 3.2 Exosome regulation by endogenous compounds

Regulation of exosomes by endogenous compounds refers to the influence that naturally occurring substances within the body have on the production, release, content, and function of exosomes. Relaxin is an antifibrotic peptide hormone. Relaxin can reduce hepatic fibrosis *in vivo*, but cannot induce activated hepatic stellate cells to quiesce *in vitro* (Hu et al., 2021). The macrophages are pivotal in the relaxin-primed alleviation of liver fibrosis *in vivo*. The macrophages secrete exosomes that promote the relaxin-mediated quiescence in activated HSCs. (Hu et al., 2021). Cleavage of fibronectin type III domain-containing protein 5 (FNDC5), a membrane-bound precursor protein, results in the formation of a myokine known as irisin. This myokine is additionally found in the liver. (Liao et al., 2023). FNDC5/Irisin has been reported inhibit the release of fibrogenic exosomes and activation of hepatic stellate cells (Liao et al., 2023). The vitamin D receptor (VDR) serves as the primary molecule that allows vitamin D to carry out its biological functions (Zhou et al., 2020). It acts as a receptor for vitamin D and is involved in various physiological processes. Activation of VDR in liver macrophages has been associated with the improvement of liver inflammation, steatosis (abnormal accumulation of fat in liver cells), and insulin resistance. This suggests that VDR activation can have beneficial effects on liver health. Vitamin D deficiency is a common finding in patients with hepatic fibrosis (Ding et al., 2013). The regulation of VDR activity is believed to be involved in the development of hepatic fibrosis (Dong et al., 2020). This indicates that VDR may play a role in the pathogenesis of hepatic fibrosis. The study confirmed that the activation of VDR was shown to alter the protein profiles within M2 macrophage exosomes (Liu et al., 2023). This change in protein content reversed the roles of these exosomes in HSC activation. The study identified Smooth muscle cell-associated protein 5 (SMAP-5) as a key effector protein within M2 macrophage exosomes that promotes HSC activation by regulating autophagy flux (Liu et al., 2023). The findings highlight the role of exosomes in mediating communication between macrophages and HSCs.

The intricate modulation of exosomes by endogenous compounds has emerged as a captivating area of investigation. By interacting with specific cellular pathways, these endogenous compounds can orchestrate the secretion of exosomes carrying specific cargo, which in turn affects target cell behavior and signaling cascades. This phenomenon holds significant implications for understanding cell-to-cell communication in health and disease, offering insights into the potential therapeutic applications of harnessing endogenous compounds to manipulate exosome-mediated processes for improved disease management and intervention strategies.

### 3.3 Exosome regulation by synthetic compounds

Control of exosome functions through synthetic compounds has garnered significant attention in recent research. These compounds offer the potential to modulate the production, cargo selection, and intercellular trafficking of exosomes, thereby influencing various physiological and pathological processes. Rupatadine (RUP) is an anti-histaminic drug with anti-oxidant and antifibrotic potential. RUP enhanced the efficacy of Mesenchymal stem cell-derived exosomes against diethylnitrosamine-induced hepatic fibrosis in rats (Didamoony et al., 2023). Carvedilol, a non-selective β-blocker, improved hepatic fibrosis in a CCL_4_ model via exosomal miR-200a enhancement (El-Wakeel et al., 2018). Pirfenidone is an anti-fibrotic drug that has been studied for its effects on hepatic fibrosis. It may influence the production and cargo of exosomes to mitigate fibrosis progression (Zhao et al., 2022). These synthetic compounds demonstrate the potential for pharmacological interventions to influence exosome functions in the context of hepatic fibrosis. By modulating the release, cargo selection, and intercellular trafficking of exosomes, these compounds offer novel therapeutic strategies to combat hepatic fibrosis.

## 4 Conclusion

While these points suggest a potential role for exosomes in hepatic fibrosis and energy metabolism, it's essential to emphasize that this field of research is still evolving. Additional studies and in-depth investigations are necessary to provide further evidence and a more comprehensive understanding of the intricate mechanisms underlying exosome-mediated regulation of energy metabolism in hepatic fibrosis. This research may have significant implications for the development of therapeutic strategies for hepatic fibrosis and related metabolic disorders. In conclusion, exosomes play a significant regulatory role in hepatic fibrosis and energy metabolism. These extracellular vesicles mediate intercellular communication and modulate energy homeostasis in hepatic cells, contributing to against the development and progression of fibrotic liver diseases. Regulating the state of HSCs by targeting exosomes may be a new idea in the development of anti-hepatic fibrosis therapies. Furthermore, exosomes hold promise as carriers for delivering hepatic fibrosis drugs. Despite the considerable diagnostic and therapeutic potential exosomes offer for hepatic fibrosis, considerable progress is required before their clinical application becomes feasible. Further understanding of the specific mechanisms underlying exosome-mediated regulation of energy metabolism will provide valuable insights for the development of novel therapeutic strategies for hepatic fibrosis.
